# Placental transfer of the polybrominated diphenyl ethers BDE-47, BDE-99 and BDE-209 in a human placenta perfusion system: an experimental study

**DOI:** 10.1186/1476-069X-9-32

**Published:** 2010-07-05

**Authors:** Marie Frederiksen, Katrin Vorkamp, Line Mathiesen, Tina Mose, Lisbeth E Knudsen

**Affiliations:** 1 Department of Environment & Health, Institute of Public Health, University of Copenhagen. Oester Farimagsgade 5, DK-1014 Copenhagen K, Denmark; 2 Department of Environmental Chemistry & Microbiology, National Environmental Research Institute (NERI), Aarhus University, Frederiksborgvej 399, 4000 Roskilde, Denmark; 3 Danish Building Research Institute, Aalborg University, Dr. Neergaards Vej 15, DK-2970 Hørsholm, Denmark

## Abstract

**Background:**

Polybrominated diphenyl ethers (PBDEs) have been widely used as flame retardants in consumer products. PBDEs may affect thyroid hormone homeostasis, which can result in irreversible damage of cognitive performance, motor skills and altered behaviour. Thus, in utero exposure is of very high concern due to critical windows in fetal development.

**Methods:**

A human ex vivo placenta perfusion system was used to study the kinetics and extent of the placental transfer of BDE-47, BDE-99 and BDE-209 during four-hour perfusions. The PBDEs were added to the maternal circulation and monitored in the maternal and fetal compartments. In addition, the perfused cotyledon, the surrounding placental tissue as well as pre-perfusion placental tissue and umbilical cord plasma were also analysed. The PBDE analysis included Soxhlet extraction, clean-up by adsorption chromatography and GC-MS analysis.

**Results and Discussion:**

Placental transfer of BDE-47 was faster and more extensive than for BDE-99. The fetal-maternal ratios (FM-ratio) after four hours of perfusion were 0.47 and 0.25 for BDE-47 and BDE-99, respectively, while the indicative permeability coefficient (IPC) measured after 60 minutes of perfusion was 0.26 h^-1 ^and 0.10 h^-1^, respectively. The transport of BDE-209 seemed to be limited. These differences between the congeners may be related to the degree of bromination. Significant accumulation was observed for all congeners in the perfused cotyledon as well as in the surrounding placental tissue.

**Conclusion:**

The transport of BDE-47 and BDE-99 indicates in utero exposure to these congeners. Although the transport of BDE-209 was limited, however, possible metabolic debromination may lead to products which are both more toxic and transportable. Our study demonstrates fetal exposure to PBDEs, which should be included in risk assessment of PBDE exposure of women of child-bearing age.

## Background

Polybrominated diphenyl ethers (PBDEs) have been widely used as flame retardant additives in a variety of products of everyday use, e.g. electric equipment, textiles and furniture upholstery. As they are not chemically bound to the polymers, they can be emitted during the product's life cycle and accumulate in the environment. With logK_ow _values of 6-7 (for tetra and penta-BDEs), they accumulate in lipid-rich tissue and biomagnify in the food chain [1]. The congeners BDE-47 and BDE-99 are among the most prevalent in the environment [1], even though their production and use was banned in large parts of the world, including the EU in 2004 [2]. Due to their persistency and toxicity, the tetra- to hepta-BDEs have also been added to Annex A of the Stockholm Convention, which aims to protect human health and the environment by eliminating toxic persistent organic pollutants (POPs) [3]. However, exposure to these compounds is likely to continue, due to the use of existing PBDE-containing products and the occurrence of BDE-47 and BDE-99 in the environment. The fully brominated congener, BDE-209, has different chemical properties than BDE-47 and BDE-99 and is less persistent in the environment despite its extreme hydrophobicity (logK_ow _≥ 10) [1]. The debromination of BDE-209 can occur by photolysis to form octa- and nonaBDEs as initial degradation products [4,5]. Furthermore, biotransformation of BDE-209 in fish and rodents results in lower brominated congeners, for example hexaBDEs [6,7] with further debromination possible. Generally, the lower brominated congeners are more toxic and bioaccumulative than BDE-209 [8,9]. In spite of its apparently lower persistence, BDE-209 has been found at very high concentrations in indoor environments, e.g., dust [10,11], as well as in humans and wildlife [12,13]. Few restrictions have been placed on the use of BDE-209, but since June 2008 the use of BDE-209 in electronic products has been banned in the EU [2]. The two US producers of BDE-209 and the largest US importer recently announced to phase out BDE-209 by the end of 2013 [14].

While PBDEs are beneficial for fire safety and save lives, they are also endocrine disrupters that interfere with thyroid hormone homeostasis [15]. The thyroid hormones are particularly important for normal brain development and impairment can result in irreversible deficits in cognitive performance and motor skills as well as altered behaviour [16,17]. In animal studies, PBDEs have been found to alter the levels of thyroid hormones in offspring after low doses of maternal exposure to PBDEs in both rodents [18] and sheep [19]. In human studies, PBDEs in house dust have been related to changes in hormone levels in men [20] and elevated levels of PBDEs have been found in breast milk of mothers to newborn boys with cryptorchidism [21].

PBDEs have been found in various human tissues, including umbilical cord blood [12,22]. The presence of PBDEs in cord blood indicates placental passage of PBDEs. In the present study, an ex vivo human placenta perfusion system was applied to investigate the transfer of the congeners BDE-47, BDE-99 and BDE-209 across the placenta. The extent of the transfer, kinetics and accumulation in the placental tissue have been compared for the three congeners. The advantage of using the human placenta perfusion model is the controlled environment in which the kinetics of the transfer can be studied and extrapolation from animals to humans is avoided.

## Methods

### Placenta perfusions

The study was approved by the regional Ethics Committee (KF 01-145/03 + KF(11) 260063) and the Data Protection Agency. All mothers gave informed written consent. Term placentas (n = 37) were collected and successful perfusions were performed on five placentas from four vaginal births and one Caesarean section directly after delivery. The placentas were injected with a Krebs Ringer buffer containing heparin (5000 IU/ml, 5 ml/l medium) to prevent any remaining blood in fetal vessels from coagulating. The perfusions were performed in a dual perfusion system as previously described [23,24]. Of the 37 placentas collected 22 were suitable for cannulation (checkpoint one in placental perfusion as described in [23]) in a chorionic artery vein pair within 30 minutes of delivery (one placenta was cannulated within 58 min, Table 1). Next, the lobule containing the perfused cotyledon was cut from the placenta (9 cm diameter) and placed in the perfusion chamber. The maternal circulation was connected by blunt cannulation of the intervillous space surrounding the perfused cotyledon. The perfusion media (100 mL each in fetal and maternal reservoirs) were circulated by peristaltic pumps and consisted of Krebs Ringer Buffer containing heparin (5000 IU/mL, Copenhagen University Hospital Pharmacy, 5 mL/L medium), glucose (0.6 g/100 mL), Pen-Strep (1% Substrate department standard solution, Panum Institute Copenhagen) and physiological levels of Human Serum Albumin (20% solution, CSL Behring Gmbh, dialysed in Krebs Ringer Buffer, maternal reservoir: 30 g/L; fetal reservoir: 40 g/L) and gassed with 95% O_2_/5% CO_2 _and 95% N_2_/5% CO_2 _in the maternal and fetal reservoirs, respectively. Of the 22 cannulated placentas only five fulfilled the criteria set for further progress in the experiments. After at least 30 minutes of pre-perfusion to stabilise temperature and oxygen content, BDE-47, BDE-99 and BDE-209 (Cambridge Isotopes Laboratories, 1 μg/mL in ethanol, 100 μL added; final concentration of 1 ng/mL) and the positive control substance antipyrine dissolved in H_2_O (Aldrich-Chemie, Germany; final concentration of 100 μg/mL) were added to the maternal reservoir. The applied concentrations of PBDEs were a compromise between achieving final levels relatively close to *in vivo *levels and having a detection frequency close to 100%. Setting the BDE-209 level was further complicated by the extremely low water solubility and generally higher blank levels than for the other congeners. The perfusions were run for four hours, and 6 mL were sampled at t = 0 from the maternal circulation and from both reservoirs before addition of the compounds and at 2, 30, 60, 130, 190 and 240 minutes after addition of the compounds. The sampled volume was not replaced.

**Table 1:** Perfusion variables.

**Parameter\Perfusion no**.	1	2	3	4	5
Volume loss, maternal (ml/min)	0.016	0.025	n.d.	0.012	0.016

Volume loss, fetal (ml/min)	0.008	0.005	0.045^a^	0.003	0.025

Flow, fetal (ml/min)	3.1	3.2	3.1^a^	2.9	3.1

Time, birth to lab (min)	n.d.	29	58	24	n.d.

Pre-perfusion (min)	48	43	56	43	48

Antipyrene, FM-ratio (at 240 min)	0.95	1.21	0.92	0.74 (1.04 at 190 min)	1.02

Maternal age (yr)	26	24	30	37	31

Placenta weight (g)	1077	675	550	620	1077

Cotyledon weight (g)	47	51	16	20	47

Total perfused (g)	113	157	86	112	113

Caesarean section	N	N	N	Y	N

Gestation age (weeks + days)	n.d.	41+3	41+0	39+1	39+4

Smoker	N	N	N	N	N

Medicine	N	N	Penicillin	Innohep & locoid one week	Citalopram

Details of donors and perfusion of five individual ex vivo placenta perfusions of PBDEs. The perfusions lasted four hours, except no. 3 for which leakage was detected after two hours.

n.d.: no data; ^a^for the first two hours only.

The pH (7.2-7.4), glucose and lactate concentrations (glucose > 6 mM), and O_2 _tension in the maternal perfusion medium (30-35 kPa), in the fetal perfusion medium (10-15 kPa), and in the fetal venous outflow (15-20 kPa) were measured every 30 minutes using an ABL5 blood gas analyser (Radiometer, Denmark). Adjustments were made using 1 M HCl or 1 M NaOH to adjust pH, glucose solution (2,61 g/L) and increasing or decreasing the rate of 95% N_2_/5% CO_2 _gassing in the fetal perfusate or 95% O_2_/5% CO_2 _gassing in the maternal perfusate to adjust pO_2 _after each measurement [23].

In addition to the perfusion samples, the three PBDE congeners were analysed in umbilical cord blood and in the placental tissue before and after the perfusion. Samples of umbilical cord blood were taken if possible and processed to collect plasma. Subsamples of the placental tissue were taken before perfusion as well as from the cotyledon and the surrounding tissue after perfusion. Prior to placenta perfusions, adsorption tests had been performed: The chambers and tubes were perfused without placental tissue to investigate loss of substance during the perfusion, for instance through adsorption to the perfusion system. These tests showed that 25-35% of BDE-47 and BDE-99 could adsorb within the perfusion system, while no loss of BDE-209 was observed.

### PBDE and antipyrine analyses

The analysis of the positive control substance antipyrine in the perfusate samples was carried out on a LaChrom HPLC system equipped with a C-18 column and a SecurityGuard precolumn eluted with methanol:water (55:45) as previously described [25].

The analysis of the PBDEs in the perfusion medium, plasma and placental tissue followed accredited methods for analysis of PBDEs in biota as previously described [13,26,27]. In brief, 5 ml of perfusate/plasma or up to 24 g homogenised placental tissue was dried with diatomaceous earth and extracted by Soxhlet using 350 mL and 500 mL hexane:acetone (4:1), respectively. The extracts were purified by multi-layer adsorption chromatography and reduced to 500 μL. Instrumental analysis was performed by GC-MS using electron capture negative ionisation (ECNI) on 60 m DB-5 and 15 m DB-1 columns.

The samples were analysed in batches of 24, which included procedural blanks corresponding to perfusate/plasma and placenta analytical methods, respectively, a duplicate analysis of one of the placenta samples and duplicate analyses of reference samples from the AMAP ring test of persistent organic pollutants in human serum. The limit of quantification (LOQ) was defined as the lowest standard of the calibration curve deviating ≤ 20% from the actual value if the compound was not detected in the blank samples. Otherwise the LOQ was set to one calibration level above the highest blank level. Thus LOQ is dependent on instrumental sensitivity at the time of the analysis and the blank level in each batch; typical LOQs were 0.005 and 0.17 ng/ml perfusate or plasma for BDE-47/-99 and BDE-209, respectively, or 0.001 and 0.06 ng/g ww for placental tissue. However, BDE-209 concentrations in the blank samples were not constant, but varied between batches.

Total lipid content in placental tissue was determined according to Smedes (1999) [28], while enzymatic lipid determination of triglyceride and cholesterol was performed on the plasma samples and total lipid was calculated as described by Covaci *et al *(2006) [29].

### Statistical methods

Student's paired t-test was used for comparison of matrices and compounds. The statistical analyses were carried out using GraphPad Prism 5.0 (GraphPad Software Inc, La Jolla, CA, USA).

## Results and discussion

### Perfusion experiments

**Figure 1:**
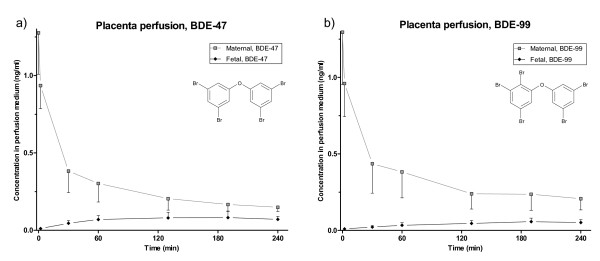
**Placenta perfusion of BDE-47 and BDE-99**. Concentration (ng/ml) and standard deviation of four hour placenta perfusions of a) BDE-47 and b) BDE-99 after addition of 1 ng/mL of each congener to the maternal compartment at t = 0. (0-60 min: n = 5; 130-240 min: n = 4).

**Figure 2:**
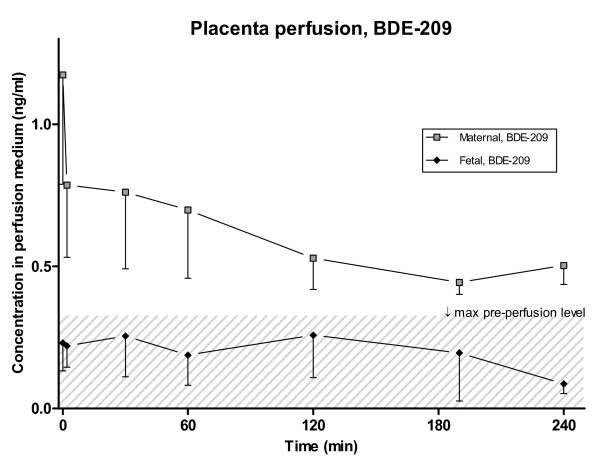
**Placenta perfusion of BDE-209**. Concentration (ng/ml) and standard deviation of four hour placenta perfusions of BDE-209 after addition of 1 ng/mL to the maternal compartment at t = 0. (0-60 min: n = 5; 130-240 min: n = 4). The grey area indicates the concentration of the maximum pre-perfusion background level in the fetal circulation.

In total, 89 samples of maternal and fetal perfusate, placental tissue and umbilical cord plasma from the five perfusions were analysed for PBDEs. The details of the five placenta perfusions are given in Table 1. The cotyledon of perfusion no. 3 was leaking towards the end of the perfusion, therefore only data from the first 60 min of this perfusion have been used. All experiments meet the success criteria on minimal leakage of fetal media (< 0.05 mL/min) and a fetal/maternal ratio (FM-ratio) for antipyrine transfer of at least 0.75 (Table 1). The oxygen transfer, glucose consumption and lactate production were monitored during the perfusion, and pH was kept in the physiological range (data not shown) [23]. It is realised that the final sampled volume of 42 ml removed from each reservoir may give a slightly different picture of transport when looking at exact values as in figures 1 and 2. As the exact same amount was removed from the fetal and maternal reservoir at the same time-points and the resulting concentration is unchanged by removal; the large sample-volume demanded by the analysis protocol for PBDE was allowed in these perfusions.

### PBDE levels

The levels of BDE-47 and BDE-99 in the maternal perfusate declined rapidly in the beginning of the perfusion; simultaneously, a significant increase in concentration was observed in the fetal perfusate (p_t(2-240), BDE47 _= 0.0003 and p_t(2-240), BDE99 _= 0.0032) (Figure 1a and 1b). The initial concentration (at t = 0) of the PBDEs in the maternal perfusate was above the expected level of 1 ng/mL that was added, which indicates insufficient initial mixing of the medium. After 2 minutes the levels were below the expected initial level. Neither BDE-47 nor BDE-99 was detected above LOQ in any of the fetal or maternal perfusate samples taken prior to the experiment.

At the end of the perfusion the average maternal concentrations of BDE-47 and BDE-99 were 0.15 and 0.21 ng/mL, respectively. This is somewhat higher than the median levels of BDE-47 found in recent studies of maternal plasma and serum in Europe (0.003-0.02 ng/mL), but within the range of recent adult median serum levels in the United States (0.09-0.4 ng/mL), assuming a lipid content of 0.8% [30]. This shows that the concentrations used in this study are within the relevant range of environmental exposure. The steady state concentrations in the fetal circulation were 0.071 and 0.052 ng/mL for BDE-47 and BDE-99, respectively, which was approximately 10 times higher than in the umbilical cord plasma from this study (Table 2), but similar to median BDE-47 levels observed in US fetal serum of 0.03-0.08 ng/mL when assuming a lipid content of 0.3% [31,32].

**Table 2:** PBDE levels in placental tissue, umbilical cord blood as well as fetal and maternal perfusate.

	BDE-47	BDE-99	BDE-209	Lipid content (%)
Cotyledon (perfused tissue) (ng/g lw)	47.9 (32.0-61.0)	73.0 (38.6-98.5)	107.8 (64.2-159.5)	1.14 (1.10-1.21)

Surrounding placental tissue (ng/g lw)	16.8 (10.7-21.04)	22.4 (16.2-27.5)	22.2 (16.3-31.8)	1.14 (1.03-1.22)

Non-perfused placental tissue (ng/g lw)	0.84 (0.57-1.16)	0.38 (< 0.11-0.75)	< LOQ	1.10 (0.95-1.34)

Umbilical cord plasma (ng/g lw)	2.37 (0.41-3.77)	1.78 (1.09-3.09)	< LOQ	0.33 (0.29-0.40)

Umbilical cord plasma (ng/g ww)	0.0078 (0.0047-0.0108)	0.0058 (0.0034-0.0089)	< LOQ	0.33 (0.29-0.40)

Fetal perfusate, t = 240 min (ng/ml)	0.071 (0.050-0.094)	0.052 (0.030-0.076)	< LOQ	n.a.

Maternal perfusate, t = 240 min (ng/ml)	0.15 (0.12-0.18)	0.21 (0.13-0.31)	0.50^a^ (0.46-0.58)	n.a.

Mean and range of PBDE levels in placental tissue, umbilical cord plasma as well as in maternal and fetal perfusate at steady state. Lipid weight basis: ng/g lw, wet weight basis: ng/g ww. (n = 4).

LOQ: Limit of quantification. n.a.: not applicable; ^a^one high outlier was removed.

The mean concentrations of BDE-47 and BDE-99 found in the non-perfused placental tissue were 0.84 and 0.38 ng/g lipid weight (lw), respectively (Table 2), and thereby in line with those previously found in Denmark and Spain [12,26]. The levels of BDE-47 were higher than those of BDE-99, which is the general trend in mammalian samples. Compared with the non-perfused tissue the concentrations were significantly higher in the tissue surrounding the cotyledon (p_47 _= 0.007; p_99 _= 0.013), and the concentrations in the actually perfused cotyledons were also significantly higher than in the surrounding area (p_47 _= 0.007; p_99 _= 0.013) (Table 2). This shows that a significant part of the amount of BDE-47 and BDE-99 added to the system accumulates in the placental tissue. After the perfusion BDE-99 was present in higher concentration in the placental tissue than BDE-47, i.e. BDE-99 accumulated in the tissue to a greater extent than BDE-47.

Contamination during analysis and thereby fluctuating blank levels are generally an obstacle in trace analysis of BDE-209 [e.g. [33]]. Blank levels are caused by the omnipresence of BDE-209, including computers and laboratory equipment, which can lead to high levels of BDE-209 in dust and indoor air, and contamination of samples and equipment in spite of careful precautions. The measured levels of BDE-209 in the non-perfused placental tissue and fetal perfusate were not significantly different from the relatively high blank levels (p_plc _= 0.3; p_fet _= 0.4), thus we chose not to report BDE-209 concentrations for these matrices (Figure 2). This critical and conservative approach is in agreement with conclusions of international PBDE intercalibration exercises [34] and the laboratory's accreditation scheme. In future perfusion studies, the use of isotope labelled BDE-209 could be considered in order to eliminate the uncertainties resulting from laboratory contamination.

In the maternal perfusate, a general decrease in BDE-209 concentration could be observed (Figure 2), to a level of 0.50 ng/mL after 4 hours of perfusion. This is roughly one order of magnitude higher than actual observed blood levels in recent studies from Denmark (~0.014 ng/ml) and elsewhere (~0.009-0.15 ng/ml, assuming 0.8% lipid content) [30,35]. In the present study, BDE-209 could not be determined in non-perfused placental tissue, however, in a previous study of placental tissue collected from the same hospital, a median level of 1.14 ng/g lw was found [26]. If a similar level is assumed in the present pre-perfusion samples, this will mean that during the perfusion BDE-209 accumulates in both the perfused cotyledon and the surrounding tissue with concentrations 20- and 100-times the background, respectively. This accumulation in placental tissue may explain why an increase in fetal circulation cannot be observed.

### Transport and kinetics of PBDEs

**Figure 3:**
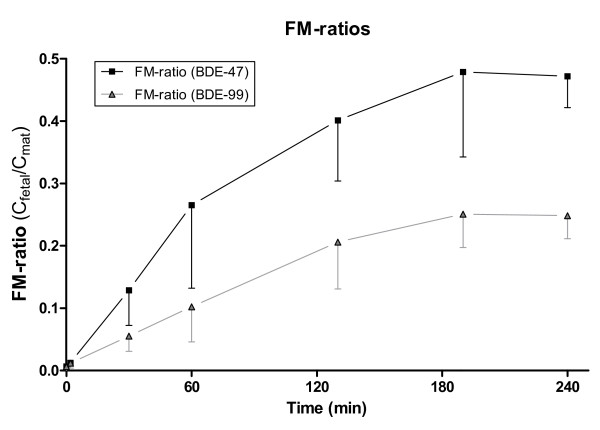
**Fetal-maternal ratios of BDE-47 and BDE-99**. Mean and standard deviation of fetal/maternal concentration ratios (FM-ratios) for BDE-47 and BDE-99 during four hour placenta perfusions. (0-60 min: n = 5; 130-240 min: n = 4).

The ratio between the concentrations in the fetal and maternal circulation (FM-ratio) during the perfusion was calculated to evaluate the extent and kinetics of the transfer (Figure 3). Steady-state was reached after approximately 190 min for both BDE-47 and BDE-99. For BDE-47 and BDE-99 the FM-ratios were 0.47 and 0.25 at steady state, respectively, thus indicating that the transfer of these PBDEs was limited. The higher FM-ratio of BDE-47 showed that it was transported across the placenta to a greater extent than BDE-99 (p = 0.0006). These results are in line with previous findings of decreasing placental transfer with increasing degree of bromination in paired maternal and umbilical cord blood [35,36]. Increasing affinity with tissue with increasing logK_OW _is a likely explanation for the decreasing placental transfer. Other compound characteristics that could affect the transport are molecular size and resulting steric hindrance [35,37] or possibly differences in affinity for carrier proteins of e.g. albumin which may control the transport of the larger congeners [38].

The rapid decrease in maternal levels indicates that BDE-209 is either transferred across the placenta or accumulated in the tissue. While BDE-209 has been detected quite frequently in adult blood samples [39-41] there are only few studies on BDE-209 in cord blood. Of these, some have found BDE-209 above the LOQ [12,22,42], mostly at low detection frequencies, while others have not [31,35,43]. Thus, in spite of the many samples below LOQ in several studies, results from the literature indicate that some transport of BDE-209 across the placenta occurs. In addition, it may be relevant to consider debromination products, which are both more toxic and transportable than the parent compound itself, when addressing in utero toxicity of BDE-209. However, the metabolic pathway of BDE-209 in humans is poorly understood. Furthermore, BDE-209 can be degraded to lower brominated compounds, leading to a potentially continued exposure to these more bioaccumulative and toxic compounds.

The rate of the transfer can be studied by the indicative permeability coefficient (IPC), which gives a quantitative indication of the permeability of the placenta for a given compound. IPC can be estimated from the slope of the initial linear section of the FM-ratio graph [44]. For BDE-47 and BDE-99, the initial linear slope covered the period from 0 to 60 minutes in Figure 3; from this, the IPC was calculated to be 0.26 h^-1 ^and 0.10 h^-1 ^for BDE-47 and BDE-99, respectively. This indicates a higher transfer rate for BDE-47 compared to BDE-99 (p = 0.014). For comparison, the IPC for the control substance antipyrine, the pesticide glyphosate, the alkaloid caffeine and the preservative benzoic acid were 0.94 h^-1^, 0.11 h^-1^, 1.03 h^-1 ^and 0.6 h^-1^, respectively, when perfused in the same human placenta model [44], and the IPC of the lipophilic substance benzo(a)pyrene was 0.08 h^-1 ^[45]. This places BDE-99 close to glyphosate and BDE-47 between glyphosate and benzoic acid with regards to the rate of the placental transport in spite of the very different properties, including, for example, aqueous solubility.

### Mass balances

**Figure 4:**
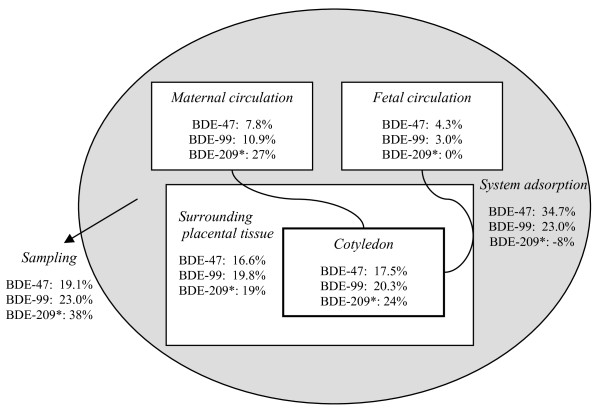
**Mass balance of BDE-47, BDE-99 and BDE-209 in the placenta perfusion system**. Fractions of originally added PBDE amounts in perfusate, placental tissue and removed by sampling. The amount unaccounted for is given as system adsorption. The numbers given for BDE-209 are indicative values (marked with *) due to possible contributions from the surroundings and higher measurement uncertainty.

A mass balance was calculated for each congener to evaluate the partitioning between the compartments at steady state using the weight or volume and the concentrations in the different compartments. The recoveries of the added compounds in the different compartments are shown in Figure 4. For BDE-47 and BDE-99, only 7.8% and 10.9%, respectively, were still in the maternal circulation at the end of the perfusion. Apart from the quantity which was removed by sampling or adsorbed to the system, the largest fraction was found in the perfused cotyledon of the placenta for both BDE-47 and BDE-99, though the amount in the surrounding tissue was almost equal but with a lower absolute concentration (Table 2). Thus, the majority of BDE-47 and BDE-99 was accumulated and recovered in the placental tissue. For BDE-47, 35% of the added amount was unaccounted for, which is in agreement with the initial system adsorption test and could reflect, for example, binding to blood cells that were removed by centrifugation prior to sample analysis. For BDE-99, 23% of the added amount was unaccounted for by tissue and media content, which is also very close to the loss observed in the system adsorption test.

No mass loss of BDE-209 was observed in the system adsorption test; however, this could be masked by the background contamination with BDE-209. The BDE-209 fractions reported (Figure 4) are indicative values where high outliers have been removed; the system adsorption was negative. This might indicate that the system has contributed slightly to the total amount of BDE-209 in the system, but could also be a result of the higher measurement uncertainty for BDE-209. As for BDE-47 and BDE-99, the majority of the BDE-209 added to the perfusion system accumulated in the placental tissue. However, a higher percentage than those of BDE-47 and BDE-99 remained in the maternal circulation after four hours of perfusion.

The mass balances do not include metabolites of the PBDEs such as hydroxyl- and methoxy-metabolites, though it is possible that differences in the metabolisation rates may account for some of the observed differences between the compounds. However, given the short time period of the study and the fact that the levels of PBDE metabolites generally are less than 10% of the parent compound in human plasma/serum [46,47] the metabolites would probably be difficult to detect with the current study setup.

### Benefits and limitations of the human placenta perfusion system

Placental passage studied in a human ex vivo placenta perfusion system provides human data non-invasively and with no major ethical concerns as the tissue is usually discarded after the birth. The setup is controlled and several compounds can be studied at the same time and can include kinetics, which is not possible to study *in vivo*. However, the method also has some limitations, for example, the necessity to exchange the blood with buffer solutions and a time limit of about six hours available for the transfer study [23]. To increase the solubility of the PBDEs in the buffer solutions, we added human serum albumin at physiological levels, and even though the study period is relatively short, the current results indicate that steady-state was obtained for BDE-47 and BDE-99 during the perfusion. Placental thickness and number of cell layers to pass from the maternal to the fetal circulation decrease towards the end of the pregnancy. Thus, the term placenta is considered more sensitive to xenobiotics than the placenta at earlier stages of pregnancy [48].

## Conclusion

To our knowledge this is the first placenta perfusion study of PBDEs. The observed transport of BDE-47 and BDE-99 across the placenta shows in utero exposure to PBDEs. The transport of BDE-47 occurred faster and more extensively than for BDE-99. In conclusions, our study clearly demonstrates fetal exposure to PBDEs, and this must be considered in risk assessments of PBDE exposure of women of child-bearing age.

## Abbreviations

AMAP: Arctic Monitoring and Assessment Programme; BDE-47: 2,2',4,4'-tetrabromo diphenyl ether; BDE-99: 2,2',4,4',5-tetrabromo diphenyl ether; BDE-209: decabromo diphenyl ether; ECNI: Electron capture negative ionisation; FM-ratio: Fetal/maternal ratio; GC-MS: Gas chromatography mass spectrometry; HPLC: High performance liquid chromatography; IPC: Indicative permeability coefficient; LOQ: Limit of quantification; PBDE: Polybrominated diphenyl ether; POP: Persistent organic pollutant.

## Competing interests

The authors declare that they have no competing interests.

## Authors' contributions

MF participated in the data analysis and drafted the manuscript. KV was responsible for the PBDE analyses and contributed to experimental planning, data analysis and reviewing of the manuscript. LM performed the perfusion experiments, antipyrine analyses and contributed to data analysis and drafting of the manuscript. TM contributed to initial planning of the perfusions and the review of the manuscript. LK was responsible for the overall study including ethical issues and contributed to experimental planning, data analysis and reviewing of the manuscript. All authors read and approved the manuscript.
